# The effect of schizotypy on spatial learning in an environment with a distinctive shape

**DOI:** 10.3389/fpsyg.2022.929653

**Published:** 2022-07-29

**Authors:** Stephanie A. Menjivar Quijano, Cameron A. Ryczek, Murray R. Horne

**Affiliations:** ^1^Department of Psychology, San José State University, San Jose, CA, United States; ^2^Department of Psychology, California State University, San Bernardino, San Bernardino, CA, United States; ^3^Department of Psychology, California State University, East Bay, Hayward, CA, United States

**Keywords:** shape, non-geometric cues, positive schizotypy, cognitive disorganization, unusual experiences, schizotypy, geometry

## Abstract

In two experiments, participants completed the Oxford-Liverpool Inventory of Feelings and Experiences measuring schizotypal traits across four dimensions (unusual experiences, cognitive disorganization, introvertive anhedonia, and impulsive non-conformity). They then took part in a virtual navigation task where they were required to learn about the position of a hidden goal with reference to geometric cues of a rectangular arena or rely on colored wall panels to find the hidden goal in a square-shaped arena. Unusual experience and cognitive disorganization were significant predictors of the use of geometric cues, but no significant predictors were found for the use of wall panels. Implications to hippocampal function and the clinical domain are considered.

## Introduction

Schizotypy (Rado, 1953; Meehl, 1990) is a latent construct that reflects one’s liability toward schizophrenia and schizophrenia spectrum disorders (SSD; Lenzenweger, 2015; Sahakyan et al., 2021). Although there is much debate on the underlying structure of the construct of schizotypy (Everett and Linscott, 2015; Fonseca-Pedrero et al., 2021), there is some agreement that the measurement of schizotypy can and should be assessed on a continuum and across multiple dimensions (Claridge and Beech, 1995; Claridge, 1997; Grant et al., 2018). Among the scales that assess schizotypy, from the Schizotypy Personality Questionnaire (SPQ; Raine, 1991) and the Wisconsin Schizotypy Scales (Winterstein et al., 2011) to the Oxford-Liverpool of Feelings and Experiences (O-LIFE; Mason et al., 1995; Mason and Claridge, 2006), three common factors emerge. These factors, supported by factor analyses, include positive, negative, and disorganized traits in non-clinical populations, which have analogs to the positive, negative and disorganized symptoms described in clinical populations of people with SSDs (Cochrane et al., 2010; Fonseca-Pedrero et al., 2018; Sahakyan et al., 2021). This relationship is also evident in the cognitive deficits that are prevalent in both SSDs and schizotypy.

Schizophrenia spectrum disorders and schizotypy produce deficits in many cognitive domains that have been categorized into either social or physical cognitive deficits. On one hand, there is evidence of deficits in how individuals process and interact with their social environment including emotional processing, theory of mind, social perception, and attributional bias (Miller and Lenzenweger, 2012; Cohen et al., 2015; Healy et al., 2016; Green et al., 2019; Dawes et al., 2021). On the other hand, and more relevant to the current study, physical cognitive deficits (Ettinger et al., 2015; Green et al., 2019) have also been shown and are considered main features of SSDs that are worth targeting for early intervention strategies (Gold, 2004; Seidman and Mirsky, 2017). Physical cognitive deficits include deficits in basic attentional processes (Catalano et al., 2021; Karamaouna et al., 2021), speed of processing (Laere et al., 2018; Gilleen et al., 2020), working memory (Forbes et al., 2009; Koychev et al., 2010; Mayer et al., 2012), and visuospatial learning (Hazlett et al., 2014; Wannan et al., 2019; Daniell et al., 2021). However, in spatial navigation (also a part of physical cognition), it is unclear if schizotypy affects spatial navigation in a similar way to SSDs. There has been many studies on the deficits in spatial navigation in SSDs (Hanlon et al., 2006; Weniger and Irle, 2008; Folley et al., 2010; Spieker et al., 2012; Wilkins et al., 2013; Mohammadi et al., 2018; Kargar et al., 2019), but only one such study involving schizotypy (Garcia-Montes et al., 2014). Therefore, the primary aim of the current set of experiments was to assess the effects of schizotypy on a virtual spatial navigation task to verify the results of Garcia-Montes et al., increase the generality of their results, and further elucidate the relationship between schizotypy and SSDs and how it relates to spatial cognition.

Patients with SSDs continuously demonstrated learning deficits in cognitively representing the relationships between multiple objects from their environment and using such relationships to find a hidden goal (i.e., allocentric coding) in a variety of virtual navigation tasks including a virtual Morris water task (Hanlon et al., 2006; Folley et al., 2010), a virtual radial arm maze (Spieker et al., 2012; Wilkins et al., 2013), and navigating within a virtual cityscape (Weniger and Irle, 2008; Mohammadi et al., 2018; Kargar et al., 2019). Few of the above studies found any significant correlations with symptomology with the exception of Folley et al. and Mohammadi et al. who found that negative symptoms were associated with deficits in allocentric navigation. However, when people with SSDs navigated through similar environments that required the use of a sequence of right- and left-hand turns or following a single object (i.e., egocentric coding), some found spared navigation ability (Hanlon et al., 2006; Weniger and Irle, 2008; Folley et al., 2010; Wilkins et al., 2013) while others found that they were impaired (Siemerkus et al., 2012; Mohammadi et al., 2018; Kargar et al., 2019). Thus, the nature of the spatial navigation deficit remains unclear, but it may be due to the focus on trying to explain the behavior as deficits in allocentric and/or egocentric strategies. In reality, studies use a variety of different navigational tasks where the strategy that is chosen and used by participants is ambiguous.

There are notable concerns with focusing on the dichotomy between allocentric and egocentric coding strategies particularly when comparing across studies. First, individuals can solve allocentric tasks using an egocentric strategy. For example, White (2008) argued that during active navigation, a person has the potential to form both stimulus-stimulus (S-S) and stimulus-response (S-R) associations, which highlight the difference between allocentric and egocentric strategies, respectively. Isolating allocentric from egocentric strategies may be problematic without using a latent learning paradigm (see Horne et al., 2012) where the development of S-R associations are prevented by not having the subject explore the environment. Second, an apparent allocentric task can be conceptualized as egocentric depending on which spatial learning theory is considered. Cognitive map theory (Tolman, 1948; O’Keefe and Nadel, 1978) suggests that one builds a mental-map of the environment by using an allocentric processing system. Alternatively, Cartwright and Collett (1983; see also Sturzl et al., 2008) suggests one takes a mental snapshot of the goal location and compares it to what is seen during navigation; minimizing errors between the two to find the goal. Snapshot theories are conceptualized as egocentric. Inconsistent findings in the SSD spatial literature could be attributed to investigating the allocentric-egocentric strategies rather than investigating potential deficits in learning different classes of stimuli. Therefore, the current experiments assessed the use of environmental geometric cues and non-geometric cues to find a goal location.

The relationship among different objects used in allocentric navigation can be characterized in two ways. First, a hidden goal can be found with reference to the individual objects and their relationship to other objects (Tolman, 1948; O’Keefe and Nadel, 1978; Suzuki et al., 1980). Second, Cheng (1986) put forward a much different conceptualization. He suggested a dedicated geometric module that encodes metric properties (angles, lengths, and left/right discriminations) independently from what he referred to as the featural subsystem which included information about landmarks (e.g., color, odor, texture, etc.). It is thus possible that the apparent deficits shown in people with schizophrenia may have more to do with participants’ inability to use the geometric arrangement of cues in their environment rather than the relationship among specific objects (for theoretical perspectives on geometry learning see Cheng and Newcombe, 2005; Cheng et al., 2013).

To our knowledge only a single study has been conducted to assess a high schizotypy sample on spatial learning. Garcia-Montes et al. (2014) had high and low schizotypal participants complete a virtual task that consisted of a virtual room that contained a 4 × 4 grid of boxes. Participants were required to find five rewarded boxes each trial and the position of the rewarded boxes did not change with respect to the room. Each participant completed 15 trials. They found no differences in latencies to task completion and errors made between high and low schizotypal subjects. There are a few concerns of this study worth mentioning. First, a test trial, in the absence of reinforcement, was not conducted to assess performance. A test trial may be more sensitive to group differences than just latencies or choices and may explain their null result. Second, they only used a categorical approach to assess the effects of schizotypy, meaning that they created high and low groups with respect to a criterion that was a combination of multiple dimensions of schizotypy. Although this method is not wrong, and perhaps goes in line with the evidence of the taxonicity of the latent structure of schizotypy (Everett and Linscott, 2015), information gained is minimized with respect to which traits may contribute to the observed effect.

The schizotypal traits that may be contributing to the predicted spatial navigation deficit in the general population are difficult to ascertain given the limited research in this area. However, the domain of attention and the neurobiology of schizophrenia may shed some light on this matter. Granger et al. (2012) assessed the effect of different schizotypal traits on an overshadowing task, which is used to assess selective attention. Participants were required to select the correct vertex on a polygon that was a combination of two triangular shapes. One triangle was larger (i.e., more salient) than the other and both shared a common side and thus two vertices, one of which was the correct vertex during training. During a test, only the smaller triangle was present and participants again had to determine which vertex was correct. A control group was included that only received training with the smaller triangle and tested in the same way. Overall, Granger et al. found that the presence of the larger triangle restricted learning about the smaller triangle (i.e., overshadowing). However, the positive schizotypy trait predicted the degree of overshadowing. There was a significant negative relationship such that as scores on the positive trait increased; the less was the overshadowing effect. The observed effect was similar in latent inhibition designs (another test of selective attention; Gray et al., 2002, 2003; Kumari and Ettinger, 2010; Kaplan and Lubow, 2011; Granger et al., 2012). Although the above experiment is not spatial navigation *per se*, participants were required to learn some aspect of geometry (e.g., angles of the vertices). Additionally, the neurobiology of geometry learning is well known. In rodents, hippocampus lesions disrupt their ability to use geometric cues (McGregor et al., 2004; Pearce et al., 2004; Jones et al., 2007). Furthermore, Sutton et al. (2012) suggested that the hippocampus might also contribute to reorientation with respect to geometric cues using fMRI in humans. This supports what we know about the morphological structure of the hippocampus in people with schizophrenia. Patients with schizophrenia have bilaterally reduced volumes in the hippocampus (Csernansky et al., 1998, 2002; Tepest et al., 2003). In addition, deformities in the CA1 region of the hippocampus was positively correlated to the severity of the positive symptoms of schizophrenia (Zierhut et al., 2010), and bilateral CA2 and CA3 volumes were negatively correlated with positive symptoms as measured by the Positive and Negative Syndrome Scale (PANSS; Kuhn et al., 2012). Individuals high on the positive trait of schizotypy show similar deficits to those with schizophrenia. Similarly, Sahakyan et al. (2021) found reduced hippocampal volumes in a sample assessed for schizotypy when compared to healthy controls. Noting the volume reduction was associated with positive and disorganized schizotypal traits. With similar hippocampal volumes, these findings further support similarities in pathology of cognitive impairment in schizotypy and schizophrenia.

In the current set of experiments, participants took the Oxford-Liverpool Inventory of Feelings and Experiences (O-LIFE) to assess schizotypy and then were required to complete a virtual navigation task on a computer. In Experiment 1 (Geometric Task), participants were required to find an unmarked goal using geometric cues of a rectangular environment. In Experiment 2 (Non-Geometric Task), participants learned that the goal was located near a specific colored wall panel in a square arena. We used a multidimensional approach to the analysis of the data to attempt to characterize the effects with respect to individual dimensions. With reduction to hippocampal volume in schizotypy and the role the hippocampus has on geometric encoding, we predict a negative relationship between performance on the geometric task and the positive traits of schizotypy (unusual experience on the O-LIFE). No traits are expected to be significant predictors of performance on the non-geometric task.

## Materials and methods

### Participants

#### Experiment 1 (geometric task)

One-hundred thirty-six undergraduate students were recruited from the Psychology Department’s subject pool at California State University, East Bay, and received course credit for their participation. Five participants were removed from the analysis for not fully completing the spatial task. The remaining 131 participants (82 females) ranged in age from 18 to 34 (M = 19.88, SD = 2.29) years of age. For transparency, participants reported the number of hours per week playing video games (M = 3.02, SD = 5.76), but this was not included in any analysis because it is highly skewed toward male participants (M_MALES_ = 6.48, M_FEMALES_ = 0.95) and not expected to affect the results in any way. No other demographic characteristics or inclusion/exclusion criteria were recorded.

#### Experiment 2 (non-geometric task)

Ninety-three participants were recruited as described in Experiment 1. Eight participants were removed from the analysis for not fully completing the task. The remaining 85 participants (56 females) ranged in age from 18 to 42 (M = 20.76, SD = 3.73) years of age and spent on average 3.49 (SD = 6.54) hours per week playing video games. As in Experiment 1, hours playing video games was not included in the analysis as it once again was skewed heavily toward males participants (M_MALES_ = 7.93, M_FEMALES_ = 1.20). No other demographic characteristics or inclusion/exclusion criteria were recorded.

### Materials

The Oxford-Liverpool Inventory of Feelings and Experiences (O-LIFE; Mason et al., 1995; Mason and Claridge, 2006) was used to measure schizotypal traits in the general population. This questionnaire measures schizotypy across four distinct dimensions: unusual experiences (UnEx; α = 0.89), cognitive disorganization (CogDis; α = 0.87), introvertive anhedonia (IntAnh; α = 0.82), and impulsive non-conformity (ImpNon; α = 0.77). UnEx reflect the positive symptoms of schizophrenia including magical ideation, IntAnh represents the negative symptoms of schizophrenia including problems with social situations and intimacy, and CogDis reflects the disorganized thoughts prevalent in people with schizophrenia. The ImpNon trait lacks evidence for a clinical analog (see Cochrane et al., 2010 for a discussion on this topic).

All virtual environments were constructed, compiled and displayed using MazeSuite software (Ayaz et al., 2008, 2011). The software ran on a desktop computer using Microsoft Windows 7 with a 2.93 GHz processor with an LCD monitor (29.2 cm × 44.5 cm). For ease of exposition, all dimensions are reported in maze units (mu) where 1 mu is approximately equal to 1.15 m and all colors are described using a 0–255 RGB (Red, Green, and Blue) scale included with the software. The same software was used in other navigation experiments with similar parameters (see Buckley et al., 2014).

#### Experiment 1: geometric task

**FIGURE 1:**
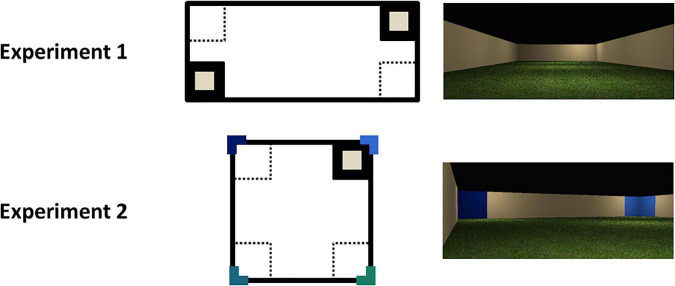
Schematic and photographs of the virtual environments used in the two experiments. The small gray squares represent the goal locations; the colored L-shapes represent the colored wall panels. The solid black and dotted squares represent the correct and incorrect zones, respectively, for the purpose of analysis.

Two environments were constructed. First, a regular octagon with 10 mu length with beige colored (204, 178, and 127) walls was created. The floor was a green grass texture and the ceiling was black (0, 0, and 0). Second, a rectangular environment (10 mu × 30 mu; see Figure 1) was made. The colors of the walls, floor and ceiling were the same as in the octagon. All environments were viewed from a first-person perspective.

#### Experiment 2 (non-geometric task)

The materials were the same as in Experiment 1, except that the experimental trials took place in a square environment (17.32 mu × 17.32 mu; see Figure 1). At each corner of the square, there were two colored wall panels (one on each wall forming the corner) that were 2 mu wide and spanned from the floor to ceiling. The panels in a single corner were the same color, but the color differed at each corner, making a total of four different colors. The colors were all RGB shades of blue-green: 51, 102, 204; 0, 25, 102; 25, 102, 127; and 25, 127, 102. All other details omitted were the same as Experiment 1.

### Procedure

All participants signed a standard consent form, and then completed the O-LIFE. Participants were then given written and verbal instructions explaining the requirements for the virtual task. The instructions conveyed to the participants the number of trials to be expected, how to move around the environments with the keyboard and a statement explaining that they may find it difficult to find the goal at first, but there is a way to find the goal on each trial. They were encouraged to explore the arena fully to get a better understanding of the environment. All procedures were approved by the institutional review board at California State University, East Bay.

#### Experiment 1 (geometric task)

The navigation task consisted of 39 trials. Trials 1 and 2 were practice trials conducted in the octagon where participants started in the center of the room facing a random orientation. The aim of the first two practice trials was to familiarize participants with using the keyboard to move around the environment. The up and down arrow moved the participant forward and backward, respectively, and the right and left arrows turned them to the right and left. Each trial lasted 30 s and after the first trial, participants were prompted to press the Enter key to begin the next trial.

Trials 3–38 were experimental trials conducted in the rectangle. Participants started in the center of the environment facing a random orientation and were required to find one of two unmarked goal locations. Each goal was a 2 mu × 2 mu area on the ground and when entered, would end the trial. A congratulatory message would appear prompting participants to press the Enter key to continue to the next trial. Each goal was situated in diagonally opposite corners (i.e., geometrically equivalent corners) where the center of the goal was positioned 2.83 mu on an imaginary line that bisected the corner. For 64 participants, the goals were located in the two corners where the long wall was to the left of the short wall. For 67 participants, goals were situated in the two corners where the long wall was to the right of the short wall.

Lastly, Trial 39 was a test trial conducted in the same manner as the experimental trials, except we removed the goals from the environment. Participants explored the environment for 60 s and then prompted that the experiment was completed. Participants received a standard debriefing statement prior to leaving.

#### Experiment 2: non-geometric task

The procedure was identical to Experiment 1 with some exceptions. The experimental trials took place in the square with different colored panels in each corner. The goal was located in the corner situated by the same colored panels across all trials. For 21, 23, 19, and 22 participants, the goal was near the landmark with an RGB of 25, 127 102; 51, 102, 204; 0, 25, 102; and 25, 102, 127, respectively. Apart from the correct colored corner, the remaining colored panels on each corner varied across trials such that there were six different possible arrangements, each used six times in a randomized fashion.

### Data preparation and statistical analyses

For both experiments, the latency to find a goal and the first corner chosen were recorded. A participant’s choice was recorded when they entered a square area of 4 mu × 4 mu located in each of the corners. Their choice was marked correct if the chosen corner contained the goal during experimental trials. It was marked incorrect for a choice to any of the remaining corners that did not contain the goal during experimental trials.

A zone analysis was conducted for the test trial in order to assess behavior in the absence of reinforcement (i.e., the goal). Each zone was a square (4 mu × 4 mu) area located at each corner. Time spent in each zone was recorded. For Experiment 1, the correct zone refers to the combined time spent in the two zones where the goals were located during experimental trials. For Experiment 2, the correct zone refers to the area of the environment that contained the goal during the experimental trials. People tended to lose motivation during the full 60 s test trial thus to get a more sensitive measure of learning, and for the purpose of analysis we looked at the first 30 s only.

Multiple linear regression models using the UnEx, CogDis, and IntAnh dimensions of the O-LIFE were used as predictors on latencies, choices, and the test trial for both experiments. All analyses were performed using SPSS with a Type I error rate of 0.05. The ImpNon dimension was omitted from all analyses as there is no agreement that impulsive non-conformity is a reliable symptom of SSDs (Cochrane et al., 2010; Mason, 2015; Thomas et al., 2019).

## Results

### Descriptive and correlations of the Oxford-Liverpool of Feelings and Experience dimensions

Tables 1, 2 show descriptive statistics and Pearson correlations for the dimensions on the O-LIFE in both experiments, respectively. Visual inspection of each of the distributions from each dimension confirmed to be normally distributed. The strengths and directions of the bivariate correlations reflected those obtained by Mason and Claridge (2006).

**TABLE 1:** Descriptive statistics for the three dimensions on the Oxford-Liverpool Inventory of Feelings and Experiences (O-LIFE) included in the analyses.

	Experiment 1: geometric task			Experiment 2: non-geometric task		
	Range	Mean	Standard deviation	Range	Mean	Standard deviation
UnEx	0–25	11.89	6.29	0–27	11.13	6.16
CogDis	2–24	12.55	5.39	0–23	12.21	5.45
IntAnh	1–19	6.68	3.83	1–16	6.46	3.84

UnEx, unusual experiences; CogDis, cognitive disorganization; IntAnh, introvertive anhedonia.

**TABLE 2:** Pearson correlations of the three dimensions on the Oxford-Liverpool Inventory of Feelings and Experiences (O-LIFE) included in the analyses.

	Experiment 1: geometric task				Experiment 2: non-geometric task			
	UnEx	CogDis	IntAnh	ImpNon	UnEx	CogDis	IntAnh	ImpNon
UnEx	—	0.51*	0.017	0.42*	—	0.50*	0.12	0.51*
CogDis	—	—	0.31*	0.19*	—	—	0.26*	0.37*
IntAnh	—	—	—	–0.05	—	—	—	–0.15

UnEx, unusual experiences; CogDis, cognitive disorganization; IntAnh, introvertive anhedonia.

*Statistically significant correlation at the 0.05 level.

### Performance

#### Experiment 1 (geometric task)

As the training progressed, participants got faster at finding the goal and made more correct choices. A dependent *t* test on the average of the first eight and the average of the last eight experimental trials was conducted and revealed a significant difference for latencies, *t* (130) = 7.71, *p* < 0.001, and choices, *t* (130) = −9.11, *p* < 0.001. For the test trial, participants spent a greater percentage of time in the correct zones than what would be predicted by chance (10.7%) based on the relative area of the arena (300 mu^2^) and correct zones (32 mu^2^). This was confirmed by a one sample *t* test on the percentage of time spent in the correct zone compared to 10.7%, *t* (130) = 23.82, *p* < 0.001.

#### Experiment 2 (non-geometric task)

As the training progressed, participants got faster at finding the goal and made more correct choices. A dependent *t* test on the average of the first eight and the average of the last eight experimental trials was conducted and revealed a significant difference for latencies, *t* (84) = 7.61, *p* < 0.001, and choices, *t* (84) = −9.49, *p* < 0.001. For the test trial, participants spent a greater percentage of time in the correct zone than what would be predicted by chance (5.3%) based on the relative area of the arena (300 mu^2^) and correct zone (16 mu^2^). This was confirmed by a one sample *t* test on the percentage of time spent in the correct zone compared to 5.3%, *t* (84) = 17.33, *p* < 0.001.

### Regressions

Tests to see if the data met the assumption of collinearity indicated multicollinearity was not a concern among the predictors for Experiment 1 (UnEx, Tolerance = 0.72, VIF = 1.40; CogDis, Tolerance = 0.65, VIF = 1.55; IntAnh, Tolerance = 0.88, VIF = 1.14) and Experiment 2 (UnEx, Tolerance = 0.75, VIF = 1.34; CogDis, Tolerance = 0.71, VIF = 1.42; IntAnh, Tolerance = 0.93, VIF = 1.07).

#### Experiment 1 (geometric task)

A Multiple linear regression was conducted on the average latency of the last eight experimental trials using UnEx, CogDis, and IntAnh, as predictors. See “Supplementary material” for the results that include the impulsive nonconformity dimension in the regression models. This analysis revealed that the three dimensions of schizotypy accounted for a significant amount of the variability in latency, R^2^ = 0.11, F (3,127) = 5.26, *p* = 0.002. The regression equation for participants predicted latency was equal to 3.61 + 0.04 (UnEx) + 0.32 (CogDis) + 0.16 (IntAnh). Only CogDis was a significant predictor of latency (*t* = 2.58, *p* = 0.011). The remaining predictors were not significant (ts < 1.09, ps > 0.05).

The same analysis was conducted with the average of the last eight experimental trials of choices and it revealed that the three dimensions accounted for a significant amount of the variance in choices, R^2^ = 0.078, F (3, 127) = 3.5, *p* = 0.016. The regression equation for participants predicted probability of making a correct choice was equal to 1.04 − 0.009 (UnEx) + 0 (CogDis) + −0.01 (IntAnh). Only UnEx was a significant predictor of choice (*t* = −2.24, *p* = 0.03). The remaining predictors were not significant (ts < 1.68, ps > 0.05).

For the test trial, an identical analysis was conducted with time spent in the correct zone as the dependent variable and it revealed that the three dimensions accounted for a significant amount of the variance in time spent in the correct zone, R^2^ = 0.07, F (3, 127) = 3.212, *p* = 0.025. The regression equation for participants predicted time spent in the correct zone was equal to 12.216 − 0.15 (UnEx) + 0.01 (CogDis) − 0.19 (IntAnh). UnEx was a significant predictor of time spent in the correct zone (*t* = −2.15, *p* = 0.03). The remaining dimensions were not significant predictors (ts < 1.76, ps > 0.05).

#### Experiment 2 (non-geometric task)

The same analyses were conducted on the latencies, choices and test trial. No regressions were significant, Fs (3, 81) < 2.64. ps > 0.05.

## General discussion

### Schizotypy and geometry learning

The purpose of the set of experiments was to explore the relationship between performance on a geometric task and schizotypy traits. Our findings revealed that the positive (UnEx) and the disorganized (CogDis) traits contributed to an individual’s ability to learn about geometric cues. A particularly strong indicator of one’s ability to learn about geometry was the UnEx dimension in both the probability of correct choices made and time spent in the correct zone during the test trial. From previous studies, the positive trait has been negatively correlated with attentional tasks such as overshadowing, and latent inhibition (Granger et al., 2012; Schmidt-Hanson and Honey, 2014; Haselgrove et al., 2015). However, this is the first demonstration of these effects in a virtual navigation task.

To a lesser extent, CogDis was a predictor of latency to find the goal during training, in that higher scores on this trait were related to slower latencies to complete a trial. Since this trait was not a predictor in any of the accuracy measures of our task we are hesitant to make any strong conclusions about its role in geometry learning. There are multiple factors that could influence one’s speed to complete a trial; motivation being a potential main factor and thus, this dependent measure may have nothing to do with the individual’s internal knowledge of the location of the goal. However, CogDis was correlated moderately with IntAnh (Table 2), and previous research has shown a relationship between negative traits, and reduced motivation and hedonic response in both a sample high in social anhedonia and in the general population. In both, there was a relationship between the negative trait and reduced prefrontal cortex activation; an area of the brain that is responsible for motivation and decision-making (Harvey et al., 2010; Hooker et al., 2014).

### Neurobiology determinants of positive schizotypy

Neurobiology may explain why UnEx was related to deficits in learning about geometric cues. As stated in the introduction, the main neural substrate involved in geometry learning is the hippocampus (McGregor et al., 2004; Pearce et al., 2004; Jones et al., 2007; Sutton et al., 2012). In addition, people with schizophrenia have reduced hippocampus volumes, bilaterally (Csernansky et al., 1998, 2002; Tepest et al., 2003) and these have been linked to the positive symptoms of schizophrenia (Zierhut et al., 2010; Kuhn et al., 2012). It is also known that unaffected siblings show similar hippocampal deformities (Tepest et al., 2003) showing a clear genetic component. Further, Sahakyan et al. (2021) and Quide et al. (2021) found similar differences in hippocampal volumes with high positive and disorganized traits significantly predicting lower volumes in the hippocampus. These results not only further strengthens the relationship between schizotypy and SSDs but also supports the conclusions drawn from the results that performance on a geometric virtual navigation task was negatively correlated with positive and disorganized schizotypal traits, which may be mediated by hippocampal abnormalities. Without imaging data on our sample, this will remain purely speculative but seems probable given the clear evidence that currently exists.

### Clinical implications

A broader question that remains is to whether schizotypy is an indicator or risk factor for the development of schizophrenia. For instance, Kwapil et al. (2013) found that positive and negative traits of schizotypy predicted SSDs and only positive traits predicted psychotic disorders on a sample that included the general population. This is also true for a study conducted on a clinical high-risk sample (Salokangas et al., 2013). There is convincing evidence that schizotypy is indeed a risk marker for developing schizophrenia or SSDs and we suggest that a phenotypic expression of this risk could be impaired learning about geometric cues. Before this can be determined, two areas of research need to be explored. First, the investigation of geometry learning in clinically high-risk samples and patient studies needs to be conducted to confirm that the variance that is explained by a fully dimensional model increases when done on a more selective sample, as in a sample of individuals that are clinically high risk. Second, the goal of looking at schizotypy as a risk factor for schizophrenia is to be able to implement early interventions. Debbane and Barrantes-Vidal (2015) have argued that adolescent expression of schizotypal traits represent a developmental link between early risk factors and later development of psychotic disorders. Thus, there is a need for research that includes a younger sample to see whether adolescents that are at high risk show impairments in geometry learning. Finally, the current findings, along with the research that has shown people high on schizotypy had problems navigating virtual cities (Mohammadi et al., 2018; Kargar et al., 2019) underscore the real impact in one’s ability to navigate the world around them.

### Limitations

The current set of experiments were conducted on college students and did not isolate clinically high-risk groups for analysis. Any clinical implications that may be derived from these results needs to be carefully considered. It is important to note that our regression models significantly explain the variance of our dependent variables greater than a model that does have any predictors. However, the amount of variance that is explained by the model is relatively low overall. This is unsurprising considering very few people of the general population will convert to having schizophrenia or SSDs. In a clinical high-risk sample, the estimated percentage of conversion was approximately 36% when a follow up assessment was done at 3 years (Fusar-Poli et al., 2013). It would be markedly lower in the general population. The Diagnostic and Statistical Manual of Mental Disorders (5th ed.; DSM-V; American Psychiatric Association, 2013) reports a lifetime prevalence of schizophrenia to be between 0.3 and 0.7%.

In our two experiments we encountered other limitations. First, our sample consisted of university students which are known for being at a lower risk of developing SSDs (Newman et al., 1998), However, Kwapil and Barrantes-Vidal (2015) point out that even if this is the case, a sample of college students can still result in meaningful data and implications since it is a more conservative sample than one drawn from the community. A community sample would be predicted to only amplify the effects shown in the current study and others that use college samples to address risk factors for developing SSDs. Second, during the experiments we did not ask about the personal or familial history of psychotic disorders in order to exclude or to analyze group differences for those that are considered high at risk.

### Future directions

The experiments support the claim that positive traits and, to some extent, disorganized traits may contribute to the impaired use of geometric cues rather than non-geometric cues. Furthermore, participants with and without a family history of schizophrenia would provide additional information concerning the nature of these deficits and implications to theories of schizophrenia (Raine, 2006) and neurodevelopmental models. As further research sheds more light on the nature of spatial learning and the schizotypy-schizophrenia relationship, our understanding of how humans navigate and the neurobiological influences of schizotypy will enhance early interventions for treating schizophrenia.

## Data availability statement

The raw data supporting the conclusions of this article will be made available by the authors, without undue reservation.

## Ethics statement

The studies involving human participants were reviewed and approved by Institutional Review Board at CSU, East Bay. The patients/participants provided their written informed consent to participate in this study.

## Author contributions

MH was the principal investigator. CR and SMQ helped equally in data collection and analysis. All authors contributed to the article and approved the submitted version.
