# Supplementary material for: The effect of schizotypy on spatial learning in an environment with a distinctive shape
Source: Front Psychol. 2022 Jul 29;13:929653. doi: 10.3389/fpsyg.2022.929653 (PMC9373985; doi:10.3389/fpsyg.2022.929653)
Supplement: Supplementary file 1 [file Data_Sheet_1.docx]

**Supplemental Material**

**(Results with impulsive non-conformity included in regression models)**

**Results**

**Experiment 1 (Geometric Task)**

A Multiple linear regression was conducted on the average latency of the last two blocks using UnEx, CogDis, IntAnh, and ImpNon as predictors. This revealed that the four dimensions of schizotypy accounted for a significant amount of the variability in latency, R^2^ = .11, F (4,126) = 3.93, p = .005. The regression equation for participants predicted latency was equal to 3.41+.031 (UnEx) + .32 (CogDis) + .17 (IntAnh) + .04 (ImpNon). Only CogDis was a significant predictor of latency (t = 2.58, p = .011). The remaining predictors were not significant (ts < 1.84, ps > .05).

The same analysis was conducted with the average of the last two blocks of choices and it revealed that the four dimensions accounted for a significant amount of the variance in choices, R^2^ = .079, F (4, 126) = 2.70, p = .034. The regression equation for participants predicted probability of making a correct choice was equal to 1.03 -.01 (UnEx) + 0 (CogDis) + -.01 (IntAnh) + .002 (ImpNon). Only UnEx was a significant predictor of choice (t = -2.20, p = .03). The remaining predictors were not significant (ts < 1.65, ps > .05).

For the probe test, an identical analysis was conducted with time spent in the correct zone as the dependent variable and it revealed that the four dimensions accounted for a significant amount of the variance in time spend in the correct zone, R^2^ = .08, F (4, 126) = 2.64, p = .037. The regression equation for participants predicted probability of making a correct choice was equal to 12.86 -.13 (UnEx) +.009 (CogDis) - .19 (IntAnh) - .11 (ImpNon). No predictors were significant.

**Experiment 2 (Non-Geometric Task)**

An identical analysis was conducted on the latencies, choices and the probe test as in Experiment 1. No regressions were significant, Fs (4, 80) < 2.09, ps < .05.
